# Contrasting Coping Styles Meet the Wall: A Dopamine Driven Dichotomy in Behavior and Cognition

**DOI:** 10.3389/fnins.2017.00383

**Published:** 2017-07-24

**Authors:** Erik Höglund, Patricia I. M. Silva, Marco A. Vindas, Øyvind Øverli

**Affiliations:** ^1^Norwegian Institute of Water Research Oslo, Norway; ^2^Centre of Coastal Research, University of Agder Kristiansand, Norway; ^3^Centro de Ciências do Mar, Universidade do Algarve Faro, Portugal; ^4^Section for Aquaculture, Institute for Aquatic Resources, Danish Technical University Hirtshals, Denmark; ^5^Uni Environment, Uni Research AS Bergen, Norway; ^6^Institute of Neuroscience and Physiology, Gothenburg University, Sahlgrenska Academy Gothenburg, Sweden; ^7^Department of Food Safety and Infection Biology, Faculty of Veterinary Medicine, Norwegian University of Life Sciences Oslo, Norway

**Keywords:** personality, monoamines, limbic system, teleosts, cognitive flexibility

## Abstract

Individual variation in the ability to modify previously learned behavior is an important dimension of trait correlations referred to as coping styles, behavioral syndromes or personality. These trait clusters have been shaped by natural selection, and underlying control mechanisms are often conserved throughout vertebrate evolution. In teleost fishes, behavioral flexibility and coping style have been studied in the high (HR) and low-responsive (LR) rainbow trout lines. Generally, proactive LR trout show a behavior guided by previously learned routines, while HR trout show a more flexible behavior relying on environmental cues. In mammals, routine dependent vs. flexible behavior has been connected to variation in limbic dopamine (DA) signaling. Here, we studied the link between limbic DA signaling and individual variation in flexibility in teleost fishes by a reversal learning approach. HR/LR trout were challenged by blocking a learned escape route, previously available during interaction with a large and aggressive conspecific. LR trout performed a higher number of failed escape attempts against the transparent blockage, while HR trout were more able to inhibit the now futile escape impulse. Regionally discrete changes in DA neurochemistry were observed in micro dissected limbic areas of the telencephalon. Most notably, DA utilization in the dorsomedial telencephalon (DM, a suggested amygdala equivalent) remained stable in HR trout in response to reversal learning under acute stress, while increasing from an initially lower level in LR trout. In summary, these results support the view that limbic homologs control individual differences in behavioral flexibility even in non-mammalian vertebrates.

## Introduction

Adaptive and flexible behavior is of paramount importance for the ability to cope with a constantly changing environment. Despite the necessity of behavioral flexibility there is considerable individual variation in this trait, which often co-varies with other aspects of phenotypic plasticity in response to environmental perturbations (reviewed by; DeWitt and Scheiner, 2004; Coppens et al., 2010).

Comparative models are indispensable with regards to providing fundamental principles of nervous system organization in vertebrates (Striedter et al., 2014). In this context, individual differences in phenotype (animal personalities, temperaments, behavioral syndromes, or stress coping styles) have frequently been identified and utilized to reveal both proximate mechanisms and evolutionary principles (Øverli et al., 2007; de Lourdes Ruiz-Gomez et al., 2011; Martins et al., 2011; Rey et al., 2013; Tudorache et al., 2013; Millot et al., 2014). Generally, there seems to be a relationship between behavioral flexibility and other traits forming individual “personalities” or stress coping styles. Bolder and more aggressive phenotypes (proactive) are characterized by active-aggressive behavior when stressed or threatened, along with low flexibility expressed as rigid, routine-like behavior, and reduced impulse control. By contrast, shy and reactive individuals display low aggression, “freeze and- hide” behavior, enhanced behavioral flexibility, and low risk taking (Coppens et al., 2010). These behavioral contrasts and associated cognitive differences such as enhanced retention of conditioned responses in proactive individuals have been suggested to underlie selection processes promoting individual differences (Øverli et al., 2007). On the one hand, proactive animals, showing a behavior response that is more guided by expectations, may do better in stable environmental conditions. Shy, reactive individuals, which are more alert to the actual situation, may flourish under variable, and unpredictable environmental conditions. Conserved patterns in the neural substrate for this variation are the subject of this paper.

Dopamine (DA) is a neurotransmitter which is associated with learning, attention, reward, and behavior reinforcement (Berridge and Robinson, 1998; Lee et al., 2006; Lemon and Manahan-Vaughan, 2006; Jenson et al., 2015). In line with this, DA has been implicated in the capability to adjust goal-directed behavioral responses to changing situations through limbic-stratial processes (reviewed by; Klanker et al., 2013). Moreover, differences in behavioral flexibility and underlying cognitive processes have been associated with individual variation in DA neurotransmission in humans (Braver et al., 2010; Barnes et al., 2011) and rodents (Laughlin et al., 2011). Accordingly, differences in forebrain DA signaling have been suggested to underlie the cognitive differences between proactive and reactive animals (Coppens et al., 2010).

The link between behavioral flexibility and stress coping styles seems to be present throughout the vertebrate linage (reviewed by Øverli et al., 2007; Sørensen et al., 2013). This suggests that common neural mechanisms controlling behavioral flexibility can disclose phylogenic roots underlying contrasting cognitive/coping styles. However, despite recent progress in linking various aspects of neural plasticity to coping style in comparative models (Øverli and Sørensen, 2016), a different developmental pattern of the forebrain in teleost fish has been constraining previous comparative studies of the functional integrity of the telencephalon (Northcutt, 2008). Still, recent lesion studies indicate functional homologies between the limbic structures, hippocampus, amygdala, and the dorsolateral (Dl) and dorsomedial (Dm) telencephalon in teleosts (Portavella et al., 2002; Demski, 2013).

Potentially, studies of the link between cognitive differences in fish with contrasting stress coping style and DA signaling in forebrain areas with limbic functions will elucidate fundamental mechanisms underlying different behavioral phenotypes. Thus, the aim of the present study is to investigate if individual differences in the ability to change behavioral strategies are reflected in DA signaling in regions with hippocampal and amygdaloid functions in fish. To achieve this, we utilized the previously established HR/LR trout model; two strains of rainbow trout (*Oncorhyncus mykkiss*) with contrasting behavioral and physiological phenotypes (high [HR] vs. low [LR] post stress cortisol production), resembling reactive and proactive stress coping styles (Øverli et al., 2007; de Lourdes Ruiz-Gomez et al., 2011). Contrasting behavioral flexibility in these strains (see e.g., Moreira et al., 2004; de Lourdes Ruiz-Gomez et al., 2011) were further investigated by modifying a social learning avoidance paradigm developed by Carpenter and Summers (2009). Presently, after learning an escape route which was available when confronted with a bigger conspecific, fish were re-exposed to the same dominant and aggressive individual while the escape route was blocked with a transparent wall. In other words, to the experimental individual it would visually appear that the escape was available, when in reality it was blocked. The number of active, but futile, attempts to escape were taken to be inversely proportional to the degree of reversal learning occurring in a stressful and threatening situation. Behavioral responses to this reversal learning approach and concomitant changes in DAergic neurochemistry in Dl and Dm were compared between reactive HR and proactive LR individuals.

## Materials and methods

### Housing and experimental fish

The experiment was carried out at The Danish Institute for Fisheries Research Station (DTU Aqua), Hirtshals, Denmark, on rainbow trout (*Oncorhyncus mykkiss*) from the 6th generation of HR/LR strains, which had been selected on post-stress cortisol levels in response to confinement stress (Pottinger and Carrick, 1999). Furthermore, these strains have been reported to display a behavioral and physiological profile largely corresponding to the proactive (i.e., LR) and reactive (i.e., HR) coping styles described for mammals (Øverli et al., 2007; de Lourdes Ruiz-Gomez et al., 2011). HR/LR fish were reared in indoor tanks (100 × 100 × 60 cm, 600 L) in a closed recirculating freshwater system on a 12:12 light/dark photoperiod and at an ambient temperature (mean temperature at time of experiment: 13.13 ± 0.63°C). Fish were fed 3 mm dry pellets (BioMar, Denmark) corresponding to an equivalent of 1.5% of their body weight by use of belt feeders (running for 12 h). An ethics approval for the experiment in the study was not required as per the Institute for Aquatic Resources, Danish Technical University's guidelines and national regulations.

### Experimental design

**Figure 1:**
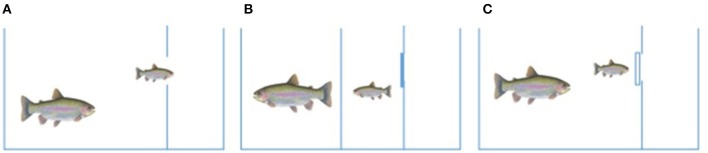
Reversal learning approach. Each aquarium consisted of one big chamber and two small chambers. The wall between the two smallest chambers had an opening (i.e., escape route) which could be opened or closed with a black or transparent door. During learning sessions, a smaller HR or LR trout interacted with a large, aggressive, and dominant conspecific (non-selected aquaculture strain) while the escape route was open **(A)**. The resident aggressive trout and the smaller HR or LR trout was separated by a removable black wall, while the escape route was closed by a back door, between training sessions, and during the acclimation phase **(B)**. During the reversal learning session, the bigger and HR or LR trout interacted while the escape route was blocked with transparent wall **(C)**.

Experimental aquaria (50 × 100 × 50 cm, 250 l) were divided by PVC walls into one 125 l compartment and two adjacent 62.5 l chambers. The wall separating the two small chambers contained an escape route (a 8.3 cm Ø opening positioned as indicated in Figure 1), while the wall separating the 125 l compartment and the middle small chamber was intact but removable. Initiating the experiment, HR and LR trout, weighing 321 ± 86 and 244 ± 51 g (mean ± standard deviation) respectively, were individually transferred to the small middle chamber of each aquarium. At the start of the experiment, the escape route was left open and the fish could move between the small chambers and familiarize themselves with the escape route. Experimental fish were considered to be acclimated when they had moved through the escape route at least two times and had displayed active feeding behavior over at least 2 consecutive days (hand feeding 0.7% of body mass and scoring feeding behavior following Øverli et al., 2006).

### Behavioral flexibility

Individual brood stock trout from an aquaculture population (not selected for stress responsiveness) weighing 1019 ± 116 g (mean ± standard deviation) were individually placed in 125 l compartments neighboring the territory of the smaller test fish 1 day before the start of avoidance learning. Behavioral flexibility was quantified with a reversal learning approach, by modification of the avoidance learning paradigm developed by (Carpenter and Summers, 2009). Upon removing the PVC walls separating small test fish and larger conspecifics, dominance-subordination hierarchies developed in the resulting size mismatched pairs. After a test fish learned to utilize the escape route available when confronted by a larger fish (Figure 1A), the smaller fish was reintroduced to the bigger fish while the escape route was blocked with a transparent PVC wall (Figure 1C). Between confrontations, small HR or LR trout were maintained in the small mid chamber with the escape route closed by an opaque PVC section (Figure 1B). To learn the escape route, the solid wall separating the small and big fish were removed and the escape route between the two small chambers was left open when HR/LR test fish interacted with their larger conspecific (Figure 1A) for seven 15 min rounds (twice daily for 3 days and one the 4th day). After these seven training rounds, the bigger fish was allowed to interact with the smaller fish for 15 min while the previously available escape route was closed with a transparent wall (Figure 1C). Half of the individuals from each strain (HR and LR) remained in isolation during this final interaction to act as isolated, non-disturbed controls. The behavior of the fish was video recorded and latency time to escape (set at 900 s, if no escape attempt) was quantified during the learning rounds. During the final session, with the escape route blocked by transparent PVC, time to initiate the first escape attempt and the number of failed escape attempts were quantified.

### Sampling procedure

All fish were anesthetized with a high dose of ethylene glycol monophenyl ether (2 ml l^−1^) until no opercular movement was observed. Fish were then weighed and brains were excised within 2 min. Brains were placed in a container with Tissue-Tek O.C.T compound (Sakura Finetek) and immediately frozen in dry ice and stored at −80°C for later brain punch micro-dissection and monoamine neurochemistry analysis.

### Dopamine analysis

Whole brains were sliced with a SLEE Cryostat MNT machine (SLEE Mainz, Germany) at −19°C in serial 300-μm sections quickly thaw mounted on glass slides, and immediately refrozen at −80°C. Micro-dissections were conducted on a BF-30 MP freezing stage for microtomes (Physitemp Instruments, USA), set at −14°C using a 337 μm Ø punch needle. The forebrain dorsolateral (Dl) and dorsomedial (Dm) pallium areas were identified using a stereotaxic atlas for rainbow trout (Navas et al., 1995). Dissected tissue samples were ejected into 100 μl of sodium acetate buffer (containing 3 g of sodium acetate, 4.3 ml of 100% glacial acetic acid and 16 sodium hydroxide pellets in 1,000 ml of Milli-Q water, the pH was corrected to 5.0 using phosphoric acid and 94.2 ng ml−1 of 3,4-dihydroxybenzyl amine hydrobromide was added, to serve as an internal standard). Samples were frozen at −80°C to facilitate cell lysis. Prior to analysis, samples were thawed on ice, and centrifuged at 17,000 rpm for 5 min. The supernatant was then removed and DA, and its principal catabolite 3,4-Dihydroxyphenylacetic acid (DOPAC) were quantified using high-performance liquid chromatography (HPLC) with electrochemical detection. The HPLC system consisted of a solvent-delivery system (Shimadzu, LC-10AD), an auto injector (Famos, Spark), a reverse phase column (4.6 × 100 mm, Hichrom, C18, 3.5 μm) and an ESA Coulochem II detector (ESA, Bedford, MA, USA) with two electrodes at −40 and +320 mV. A conditioning electrode (ESA 5020) with a potential of +400 mV was employed before the analytical electrodes, to oxidize possible contaminants. The mobile phase consisted of 86.25 mM l^−1^ sodium phosphate, 1.4 mM l^−1^ sodium octyl sulfate and 12.26 μM l^−1^ EDTA in deionized (resistance 18.2 MW) water containing 7% acetonitrile brought to a pH of 3.1 with phosphoric acid. Samples were quantified by comparison with standard solutions of known concentrations and corrected for recovery of the internal standard using HPLC software (CSW, DataApex Ltd, Czech Republic). The tissue pellet remaining from each sample was dissolved in 110 ml 0.4 N NaOH and protein content was assayed (Bradford, 1976).

### Data analysis

Number of training sessions needed to escape for the first time and numbers of failed escape attempts were compared between HR/LR genotypes by Mann–Whitney *U*-tests. DOPAC/DA ratios, DA and DOPAC concentrations in micro-dissected Dm and Dl were analysed by a two-way analysis of variance (ANOVA), with treatment (social interaction with a blocked escape route vs. isolated controls) and strain (HR vs. LR) as independent variables, followed by a Tukey–HSD *post-hoc* test when required. DOPAC/DA ratio was arcsin transformed and DA and DOPAC concentrations were log transformed to achieve normal distribution.

## Results

### Behavior

**Figure 2:**
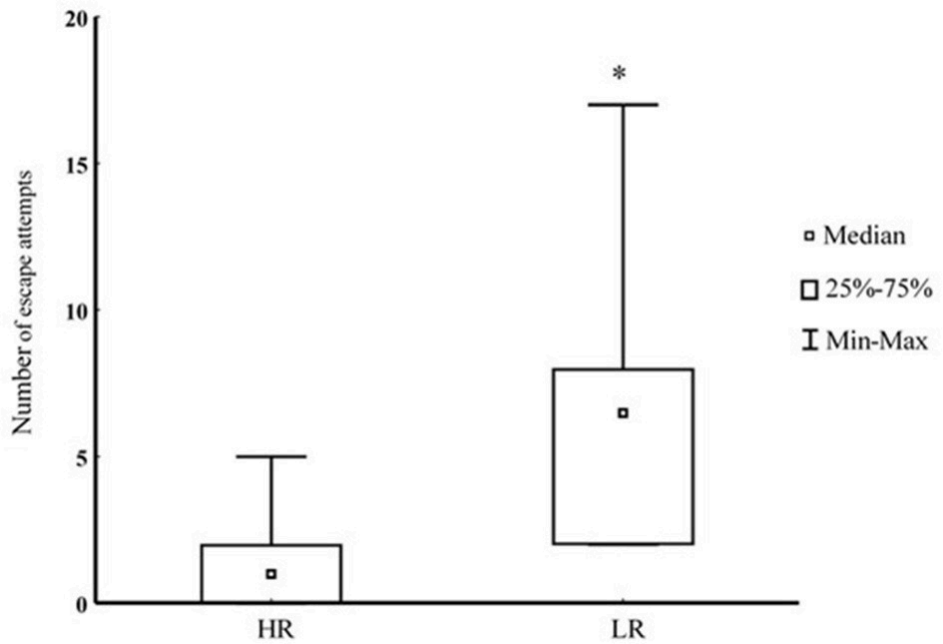
Number of escape attempts toward an escape route blocked by transparent wall during interaction with a bigger opponent in HR (*n* = 7) and LR (*n* = 6) fish. ^*^Denotes *P* < 0.05.

Prior to closure of the escape route, there were no difference in the number of training sessions needed to escape for the first time (HR 3 ± 1, LR 3 ± 1; median ± upper and lower quantile; *P* = 0.75). During the final physical encounter, when the escape route was closed, LR trout demonstrated lack of reversal learning by performing a higher number escape attempts toward the transparent wall blocking escape (*P* < 0.05; Figure 2).

### DA neurochemistry

**Figure 3:**
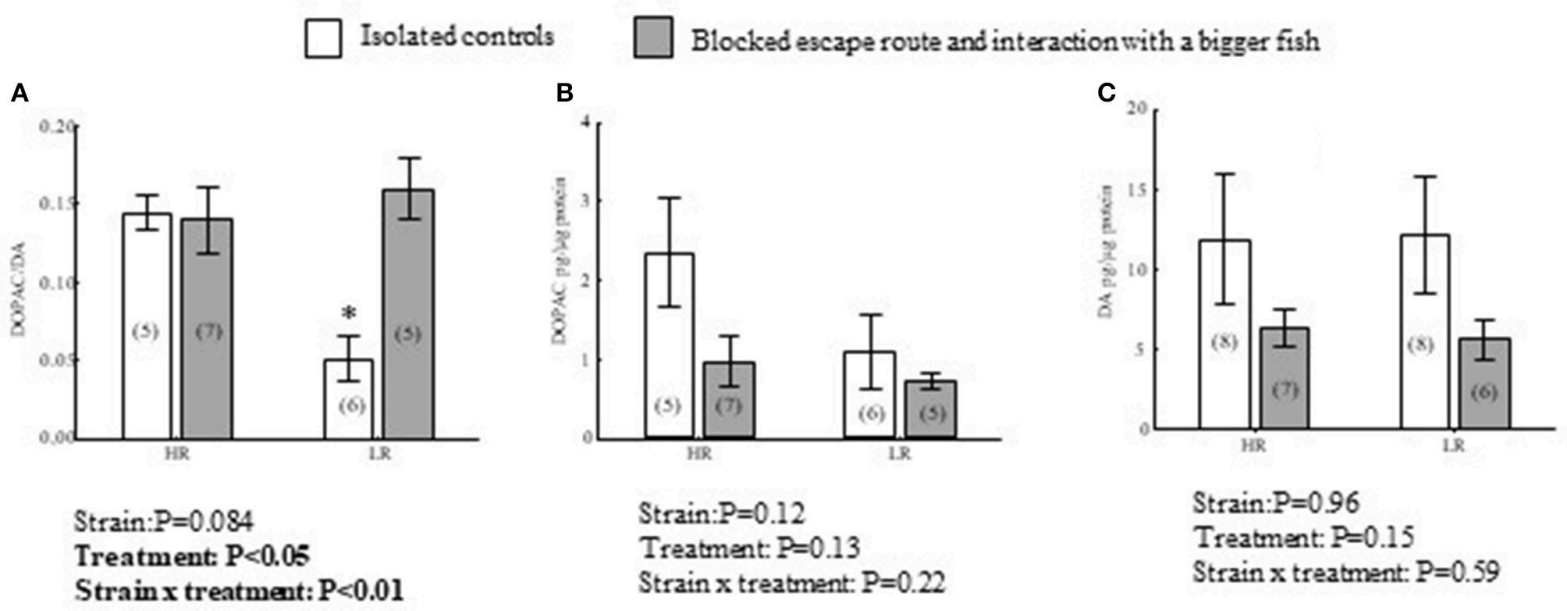
Effects of treatment, isolated control or interacting with a bigger conspecific when a transparent wall blocked a learned escape route, and strain on DOPAC/DA **(A)** ratios, and DOPAC **(B)** and DA **(C)** concentrations in the dorsomedial telencephalon (Dm) of HR and LR fish. *P*-values of two-way ANOVA statistics results are presented in figures. For complete ANOVA statistics see results. ^*^Denotes that isolated LR controls differs from interacting LR, interacting HR and isolated HR trout at a significance level of *P* < 0.05. Values within parentheses = *n*.

In Dm there were significant interaction effects between treatment and strain in DOPAC/DA ratios [*F*_(1, 19)_ = 8.7, *P* < 0.01; Figure 3A]. In LR trout, fish interacting with a bigger opponent while the escape route was closed with a transparent wall showed elevated values compared to isolated controls (*P* < 0.01). In HR trout, there were no significant differences between interacting and isolated control fish regarding this indicator of DA utilization (*P* = 0.99). Moreover, the elevated DOPAC/DA ratios in interacting LR trout reached those of HR trout. This was reflected in no significant difference between interacting LR trout, and either interacting (*P* = 0.87) or isolated HR controls (*P* = 0.96). DOPAC/DA ratios in isolated control (*P* < 0.05) and interacting (*P* < 0.05) HR trout were significantly higher compared to isolated control LR trout. In addition to the significant interaction effect on DOPAC/DA ratios, there was an overall treatment effect [*F*_(1, 19)_ = 6.4, *P* < 0.05], apparently driven by the DOPAC/DA reduction exclusive to LR fish (Figure 3A). There was no significant strain specific effect independent of treatment [*F*_(1, 19)_ = 4.7, *P* = 0.084]. However, the above differences in DA turnover were not reflected in DA or DOPAC concentrations alone (Figures 3B,C). DOPAC concentrations were subject to neither interaction effects between treatment and strain [*F*_(1, 19)_ = 1.6, *P* = 0.22], strain specific effect effects independent of treatment [*F*_(1, 19)_ = 2.6, *P* = 0.12], nor treatment specific effects independent of strain [*F*_(1, 19)_ = 2.5, *P* = 0.13; Figure 3B]. Also for DA concentrations, interaction effects between treatment and strain were not evident [*F*_(1, 25)_ = 0.33, *P* = 0.59], as with strain specific effects independent of treatment [*F*_(1, 25)_ = 2.1, *P* = 0.15] and treatment specific effects independent of strain [*F*_(1, 25)_ = 0.30, *P* = 0.59; Figure 3C].

**Figure 4:**
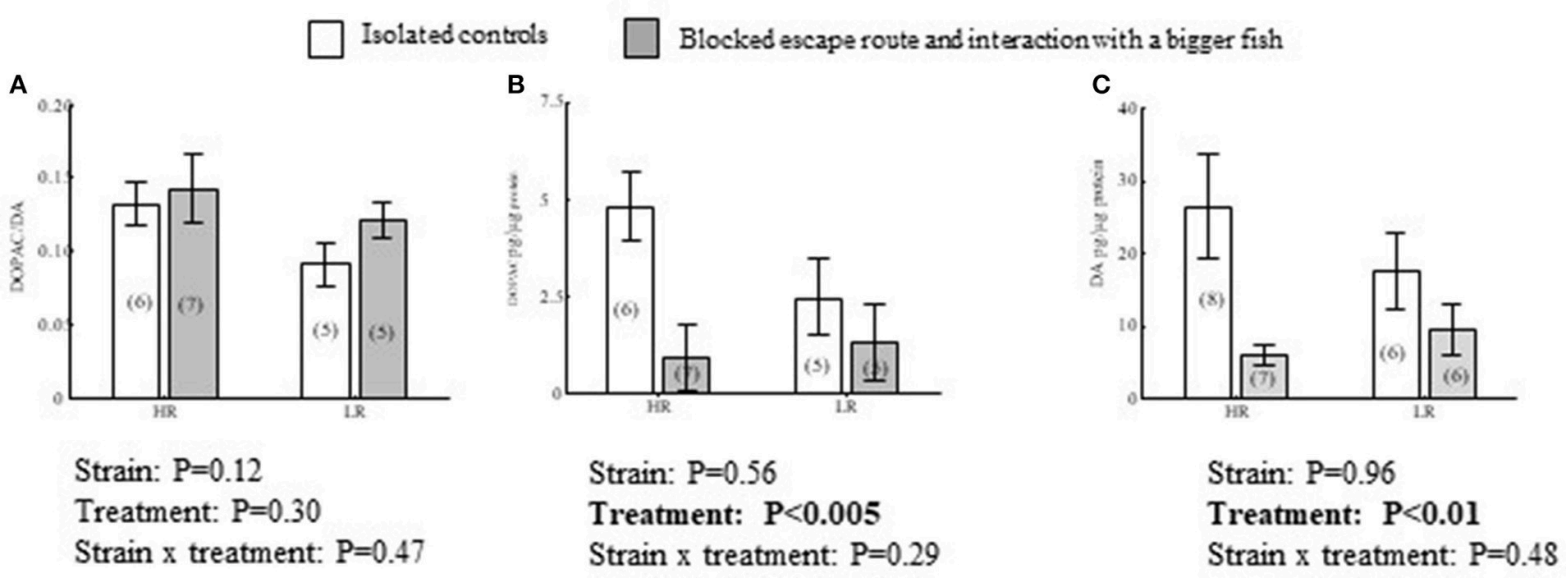
Effects of treatment, isolated control or interacting with a bigger conspecific when a transparent wall blocked a learned escape route, and strain on DOPAC/DA **(A)** ratios, and DOPAC **(B)** and DA **(C)** concentrations in the dorsolateral pallium (Dl) of HR and LR fish. *P*-values from a Two-way ANOVA are presented in figures. For complete ANOVA statistics see results. Values within parentheses = *n*.

In Dl, there were treatment effects which were independent of strain, with DA concentrations being generally higher in isolated controls than in fish exposed to larger conspecifics in combination combined with a blocked escape route [*F*_(1, 25)_ = 0.13, *P* < 0.01; Figure 4C]. The same pattern was observed in DOPAC concentrations, where a blocked escape route resulted in lower DOPAC levels compared to isolated controls [*F*_(1, 19)_ = 10, *P* < 0.005; Figure 4B]. However, concomitant changes in both transmitter and catabolite yielded no effect on DOPAC/DA ratios [*F*_(1, 19)_ = 1.2, *P* = 0.47; Figure 4A]. In Dl, there were no significant effects of strain that were independent of treatment in DA concentrations [*F*_(1, 25)_ = 0.00, *P* = 0.96], DOPAC concentrations [*F*_(1, 19)_ = 0.35, *P* = 0.54] or DOPAC/DA ratios [*F*_(1, 19)_ = 2.7, *P* = 0.12; Figures 4A–C]. Neither were there interaction effects between treatment and strain in DA concentrations [*F*_(1, 25)_ = 0.52, *P* = 0.48; Figure 4C], DOPAC concentrations [*F*_(1, 19)_ = 1.2, *P* = 0.29] or DOPAC/DA ratios [*F*_(1, 19)_ = 0.54, *P* = 0.47; Figures 4A–C].

## Discussion

In this study, the rate of acquiring use of the escape route when being confronted with a bigger conspecific was similar between HR and LR trout. This is in accordance with previous studies demonstrating no difference between the strains in learning novel tasks (de Lourdes Ruiz-Gomez et al., 2011) or acquiring conditioned memories (Moreira et al., 2004). However, in the reversal learning challenge, LR trout performed higher numbers of escape attempts toward an invisible wall blocking the learned, previously available escape route. This indicates that LR fish base their behavior on expected outcome and previously learned routines, impeding behavioral adjustment in new situations. Again, the result corresponds well with previous studies demonstrating enhanced retention of conditioned responses (Moreira et al., 2004) and lack of responsiveness to changes in the environment in proactive LR fish (de Lourdes Ruiz-Gomez et al., 2011). Such differences in cognitive flexibility are in line with a generally stronger tendency to develop and follow routines in proactive individuals also of other species (Verbeek et al., 1994; Bolhuis et al., 2004). As will be discussed below, the sudden removal of a previously available escape opportunity incurred limbic DA responses which were similar between strains/coping styles in the dorsolateral pallium (Dl, a concomitant drop in both DA and DOPAC concentrations) but contrasting in the dorsomedial pallium (Dm, increased DOPAC/DA ratios in LR but not in HR).

A general response pattern of the proactive LR strain seems to be that they have a higher threshold for noticing and/or reacting to challenges compared to the HR strain (Øverli et al., 2002). This is in line with a generally higher tendency to base behavioral responses on inherent predictions of the actual environment in proactive copers, while reactive copers show a more direct stimulus–response (Coppens et al., 2010). In mammals, the amygdala and the hippocampus have been shown to act together in detection of environmental novelty (Blackford et al., 2010). DA signaling has been associated with elevated neural plasticity in hippocampus and other brain regions. In line with this, elevated levels of other compounds providing substrates for a higher degree of behavioral flexibility (i.e., proliferating cell nuclear antigen and neurogenic differentiation factor) have been reported in proactive individuals (Johansen et al., 2012; Vindas et al., 2017). Fishes showing a generally more widespread capacity for neural plasticity than mammals, this suggests that the lower baseline DAergic activity in Dm, a region with amygdaloid functions, in isolated LR trout reflects a generally lower capability to detect and react to environmental stimuli in this strain.

The blockade of the escape route during exposure to a bigger opponent resulted in that DA-ergic activity increased in LR fish and reached levels of isolated as well as interacting HR trout. In mammals, DA release in amygdala is related to higher levels of arousal during stressful situations (Inglis and Moghaddam, 1999). This might suggest that the elevated Dm DAergic activity after interaction with a bigger conspecific while a learned escape route was blocked might reflect elevated arousal induced attention in LR trout. Rodent models of “surprise induced attention” alludes to the latter, demonstrating that DA signaling in amygdala plays an important role in increased attention generated by prediction error (Lee et al., 2006). Of note, however, is that the increase in DOPAC/DA ratio seen in LR fish during reversal learning apparently depended rather heavily on a drop in DA concentrations, thus further studies are needed to determine with certainty that the change in turnover reflects increased utilization vs. decreased synthesis.

In Dl, there were no strain specific effects, in that blocking of a learned escape route resulted in a concomitant decrease in DA and DOPAC concentration in both HR and LR trout, with DOPAC/DA ratios remaining stable as a result. Moreover, in Dm this effect of blocking the escape route on DOPAC and DA concentrations was less pronounced. Considering that DA concentrations are related to production, while DOPAC is mostly related to catabolism of released DA, this suggests that these neurochemical changes reflects a lower production of DA in Dl during the combination of acute social interaction and changed environmental parameters. In mammals, DA producing nuclei within the ventral tegmental area (VTA) and substantia nigra (SN) innervates striatal, limbic and cortical regions. However, the majority of the VTA DA cells projects to cortical and limbic regions, while the majority of the SN DA cells projects to striatal regions (for references see; Cools, 2008). Likewise, there are indications of midbrain DA producing nuclei with different projection pattern to forebrain areas in teleost fish. DA cells sited around the periventricular nucleus of the posterior tuberculum project to the Vv-Vd striatal/limbic areas and cells in posterior tuberal nucleus project to the pallillal areas of the teleost telencephalon. (Rink and Wullimann, 2002) Further studies of DA projections are needed to investigate if the less pronounced effect of a closed escape route on Dl in dopamine production/activity is related to enervation from different midbrain nucleus.

In mammals, hippocampal DA plays an important role in the process of detection and storage of unpredicted events (for references see; Lemon and Manahan-Vaughan, 2006). This implies that the elevated DA and DOPAC concentrations in Dl may reflect increased attention and act as substrates for learning in fish interacting with a bigger conspecific when the escape route was blocked. Moreover, in mammals, the amygdala and the hippocampus have been shown to act together in detection of environmental novelty (Lemon and Manahan-Vaughan, 2006). This makes it tempting to suggest that the general decrease in hippocampal DOPAC and DA concentrations in fish interacting with a bigger fish, together with the “surprise” induced elevation in amygdaloid DAactivity in LR trout, may reflect cognitive differences underlying strain contrasts in behavioral flexibility. In addition to limbic structures, mammalian studies show that DA in striatum plays a central role in cognitive processes underlying behavioral flexibility (reviewed by; Cools, 2008). Unfortunately, the DA and DOPAC in Vv, a region with striatal functions, concentrations were under the detection limit in our study. This calls for further studies of regional in forebrain DA signaling, including Vv, in fish showing contrasts in behavioral flexibility to further disclose the evolutionary roots of the link between different, individually variable, personality traits such as stress responsiveness and cognitive and behavioral responses to sudden changes in the surrounding environment.

## Ethics statement

An ethics approval for the experiment in the study was not required as per the Institute for Aquatic Resources, Danish Technical University's guidelines and national regulations. The treatment in this experiment is considered to cause less discomfort than a needle injection. At the time the experiment was conducted (2013), these types of experiment could be performed without permit in Denmark.

## Author contributions

EH finalized the manuscript and performed the data analyses. PS drafted the first version of the manuscript MV took part in data analyses and writing up the manuscript ØØ took part in data analyses and writing up the manuscript.

### Conflict of interest statement

The authors declare that the research was conducted in the absence of any commercial or financial relationships that could be construed as a potential conflict of interest.
